# Patterns of injury and outcomes in the elderly patient with rib fractures: a multicenter observational study

**DOI:** 10.1007/s00068-018-0969-9

**Published:** 2018-06-15

**Authors:** Mark G. Van Vledder, Vicky Kwakernaak, Tjebbe Hagenaars, Esther M. M. Van Lieshout, Michiel H. J. Verhofstad, On behalf of the South West Netherlands Trauma Region Study Group, Onno Boonstra, P. Ted den Hoed, Tijs Jakma, Jan L. M. van Niekerk, Piet A. R. de Rijcke, Geert R. Roukema, Vicktor A. de Ridder, Georg B. Schmidt, Marco Waleboer

**Affiliations:** Trauma Research Unit, Department of Surgery, Erasmus MC, University Medical Center Rotterdam, P.O. Box 2040, 3000 CA Rotterdam, The Netherlands

**Keywords:** Rib fractures, Geriatric trauma, Registry study

## Abstract

**Background:**

High rates of pneumonia and death have been reported among elderly patients with rib fractures. This study aims to identify patterns of injury and risk factors for pneumonia and death in elderly patients with rib fractures.

**Methods:**

A retrospective multicenter observational study was performed using data registered in the national trauma registry between 2008 and 2015 in the South West Netherlands Trauma region. Data regarding demographics, mechanism of injury, pulmonary and cardiovascular history, pattern of extra-thoracic and intrathoracic injuries, ICU admission, length of stay, and morbidity and mortality following admission were collected.

**Results:**

Eight hundred eighty-four patients were included. Median age was 76 years (P_25_–P_75_ 70–83). 235 patients (26.6%) were 81 years or older. Moderate or worse extra-thoracic injuries were present in 456 patients (51.6%), of whom 146 (16.6%) had severe head injuries and 45 (5.1%) severe spinal injuries. Median ISS was 9 (P_25_–P_75_ 5–18). The rate of pneumonia was 10% (*n* = 84). Ten percent of patients (*n* = 88) died. Risk factors for in-hospital mortality included age (OR 3.4; *p* = 0.003), presence of COPD (OR 1.3; *p* = 0.01), presence of cardiac disease (OR 2.6; *p* = 0.003), severe or worse head (OR 3.5; *p* < 0.001), abdominal (OR 6.8; *p* = 0.004) and spinal injury (OR 4.6; *p* = 0.011) by AIS, number of rib fractures (OR 2.6; *p* = 0.03), and need for chest tube drainage (OR 2.1; *p* = 0.021).

**Conclusions:**

Pneumonia and death occur in about 10% of elderly patients with rib fractures. Apart from the severity of thoracic injuries, the presence and severity of extra-thoracic injuries and cardiopulmonary comorbidities are associated with poor outcome.

## Introduction

Rib fractures do commonly occur in elderly patients (65 years and older) following blunt thoracic trauma [1]. Although these injuries are often caused by low-energy trauma (e.g., fall from standing height), adverse outcome such as pneumonia, respiratory failure and death are frequently observed [2, 3]. Immediate recognition of patients at risk for developing such adverse events after being admitted for thoracic injuries resulting from blunt thoracic trauma is, therefore, of vital importance.

Apart from risk stratification, treatment decisions regarding rib fractures may be impacted by the presence of certain patient characteristics or extra-thoracic injuries. For instance, oral anticoagulant use, the presence of severe spinal injuries or severe brain injury may preclude epidural analgesia or surgical rib fixation. Data with regard to patterns of injury among elderly patients with rib fractures are especially helpful when considering such treatment regimens.

The aim of the current study was to identify risk factors for pneumonia and death in conservatively treated elderly patients with rib fractures. Furthermore, patterns of injury were investigated.

## Patients and methods

For this retrospective observational multicenter study, patients were identified using data from the Dutch national trauma registry. This registry includes all patients admitted to any hospital in the Netherlands following any traumatic injury. Medical case records are reviewed directly after discharge and data are inserted in the national database by trained data managers. Study design, data analysis and drafting of the manuscript were performed according to the STROBE guidelines for cohort studies. The study protocol was approved by the institutional review board of the coordinating hospital.

For this study, the national trauma registry was queried for elderly patients (65 years or older) with rib fractures. Patients were included if they were admitted between January 1st, 2008 and December 31st, 2015 in one of ten different hospitals in the South West Netherlands Trauma region and had at least one rib fracture following blunt trauma. Patients were excluded from the analysis if they died within 24 h of admission due to extra-thoracic injuries, had cervical spine injury with complete paralysis of respiratory muscles, if they underwent surgical rib fixation or if they had no data in their case record. If patients were transferred to another hospital directly from the emergency room, their cases were only included in the analysis if the hospital eventually admitting the patient was one of the cooperating hospitals.

In the Dutch trauma system, each hospital is assigned a specific level of trauma-expertise. Level-1 hospitals are dedicated trauma centers equipped to take care of patients with multiple, potentially life-threatening injuries. Level-2 hospitals are equipped to receive and treat hemodynamic stable patients with multiple (non-acutely life-threatening) injuries. Level-3 hospitals generally only admit patients with single injuries that do not pose an acute threat to the patient’s life. Triage with regard to which type of hospital a patient should be transported is performed by ground emergency medical personnel, if necessary assisted by a Dutch physician staffed helicopter emergency medical services (HEMS).

The following variables were collected from the national trauma registry: age, gender, trauma mechanism, hospital length of stay, ICU admission, length of ICU admission, injury severity score (ISS), and specific injuries as coded by the abbreviated injury scale (AIS). Three AIS groups were created for each body region for easy comparison: 1 none or only minor injuries (AIS 0 or 1), 2 moderate injuries (AIS 2), 3 severe or worse injuries (AIS 3 or higher). Registry data were supplemented with data from a retrospective chart review in which the following variables were collected: presence of cardiovascular disease, presence of pulmonary disease, medication, hemo- or pneumothorax requiring chest-tube drainage, diagnosis of pneumonia (progressive or new infiltrate on chest X-ray combined with two of the following characteristics; temperature alteration < 36.0 °C or > 38.3 °C, purulent sputum or leukocytosis < 5000cells/mm^3^ or > 10,000cells/mm^3^), and late respiratory failure (> 24 h after admission) requiring tracheal intubation and mechanical ventilation.

Data were analyzed using the statistical package for the social sciences (SPSS) version 23.0 (SPSS, Chicago, Ill, USA). Missing values were not imputed. For continuous non-parametric data, the median and percentiles are reported. Distribution of continuous data was checked using the Shapiro–Wilk test for normality. For categorical data, numbers and frequencies are reported. Exploratory analysis to detect any associations between the primary and secondary outcome variables and patient, injury and treatment characteristics was performed using the non-parametric Mann–Whitney test for continuous variables and Pearson chi-square test for categorical variables. Risk factors for death and pneumonia were further investigated using univariable logistic regression analysis. Odds ratios, 95% confidence intervals and *p* values are reported. Independent variables with a statistically significant association with the outcome variable in the univariable analysis were included in a multivariable binary logistic regression model in a stepwise (backward and forward) fashion. Goodness of fit was determined using Nagelkerke *R*^2^ and the Hosmer and Lemeshow test. A *p* value of < 0.05 was considered to be statistically significant.

## Results

### Patient characteristics

**Fig. 1:**
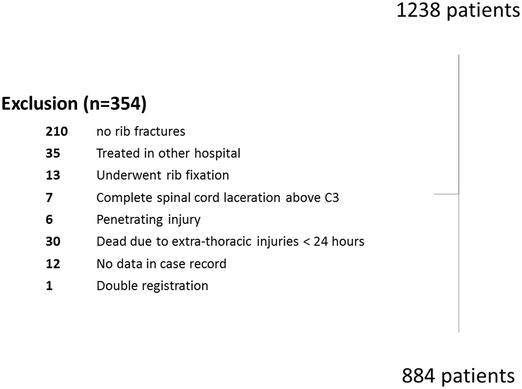
Flowchart of patients included and excluded in the analysis

A total of 1238 patients were identified in the database meeting the search criteria. After excluding 354 patients that fulfilled one or more of the exclusion criteria, 884 patients remained for further analysis (Fig. 1). Patient demographics are provided in Table 1. About two-third of patients were between 65 and 80 years old. Cardiac comorbidity was present in one-fifth of patients. Pulmonary disease was present in 10.5% of patients. The mechanism of injury was a fall from standing height in 43.7% of patients.

**Table 1:** Baseline characteristics of 884 elderly patients with rib fractures

Characteristic	Median (P_25_–P_75_)	Number (%)
Age		
Age	76 (70–83)	
Age 65–80		580 (65.6%)
Age 81–90		235 (26.6%)
Age > 90		69 (7.8%)
Gender		
Female		369 (41.7%)
Male		515 (58.3%)
History of cardiac disease		
None		711 (80.4%)
Myocardial infarction		85 (9.6%)
Congestive heart failure (any NYHA grade)		59 (6.7%)
Unknown		29 (3.3%)
Oral anticoagulation use		
Yes		151 (17.1%)
No		618 (81.2%)
Unknown		15 (1.7)
History of COPD		
None		764 (86.4%)
COPD (any GOLD stadium)		93 (10.5%)
Unknown		27 (3.1%)
Mechanism of Injury		
Low energetic fall		387 (43.7%)
High energetic fall		147 (16.6%)
Bike or pedestrian		150 (17.0%)
Motor vehicle accident		99 (11.2%)
Other		17 (1.9%)
Unknown		82 (9.3%)

An overview of patterns of injury is provided in Table 2. To account for pre-hospital triage of patients and subsequent referral to level-1, level-2 or level-3 centers, data are stratified by the level of the receiving hospital. While about a quarter of patients had four or more rib fractures, a clinically manifest flail chest was present in only 2.5% of patients. Chest tube drainage was performed in 17% of patients. Injury severity scores and the number and severity of injuries were significantly higher in the level-1 trauma center than in the level-2 and level-3 centers. Over half of all patients had moderate (23.3%), or severe or worse (28.3%) extra-thoracic injuries. In the participating level-1 hospital this was as high as 65.1% of patients. Of the 238 patients with four or more rib fractures, ninety (37.8%) patients also had severe or worse extra-thoracic injuries. More specifically, 53 patients (22.3%) also sustained severe or worse head injury and 19 patients (8.0%) also sustained severe or worse spinal injuries.

**Table 2:** Patterns of intra-thoracic and extra-thoracic injury stratified by level of admitting hospital

Characteristics	All *N* = 884	Level 1 *N* = 235	Level 2 *N* = 425	Level 3 *N* = 224	*p* value
ISS					
Median (P_25_–P_75_)	9 (5–18)	22 (16–33)	9 (5–13)	9 (4–10)	< 0.001
< 16	605 (68.4)	55 (23.4)	349 (82.1)	201 (89.7)	< 0.001
≥ 16	279 (31.6)	180 (76.6)	76 (17.9)	23 (10.3)	
Head injury					
None or minor	674 (76.2)	109 (46.4)	361 (84.9)	204 (91.1)	< 0.001
Moderate	64 (7.2)	15 (6.4)	35 (8.2)	14 (6.3)	
Severe or worse	146 (16.6)	111 (47.2)	29 (6.8)	6 (2.3)	
Abdominal injury					
None or minor	843 (95.3)	211 (89.8)	413 (97.2)	218 (97.3)	0.001
Moderate	29 (3.3)	16 (6.8)	9 (2.1)	4 (1.8)	
Severe or worse	13 (1.4)	8 (3.4)	3 (0.7)	2 (0.9)	
Spinal injury					
None or minor	774 (87.6)	157 (66.8)	404 (95.1)	213 (95.1)	< 0.001
Moderate	65 (7.4)	44 (18.7)	14 (3.3)	7 (3.1)	
Severe or worse	45 (5.1)	34 (14.5)	7 (1.6)	4 (1.8)	
Lower extremity injury					
None or minor	765 (86.5)	181(77.0)	377 (88.7)	207 (92.4)	< 0.001
Moderate	49 (5.5)	21 (8.9)	15 (3.5)	13 (5.8)	
Severe or worse	70 (7.2)	33 (14.0)	33 (7.8)	4 (1.8)	
Thoracic injury					
Minor	135 (15.3)	25 (10.6)	68 (16.0)	42 (18.8)	< 0.001
Moderate	308 (34.8)	53 (22.6)	165 (38.8)	90 (40.2)	
Severe or worse	441 (49.9)	157 (66.8)	192 (45.2)	92 (41.1)	
Extra-thoracic injuries (any)					
None or minor	428 (48.4)	39 (16.6)	248 (58.4)	141 (62.9)	< 0.001
Moderate	206 (23.3)	43 (18.3)	98 (23.1)	65 (29.0)	
Severe or worse	250 (28.3)	153 (65.1)	79 (18.6)	18 (8.0)	
Number of rib fractures					
Single	168 (19.0)	32 (13.6)	83 (19.5)	53 (23.7)	< 0.001
2–3	341 (38.6)	68 (28.9)	171 (40.2)	102 (45.5)	
≥ 4	232 (26.2)	92 (39.1)	100 (23.5)	40 (17.9)	
Multiple not further specified	143 (16.2)	43 (18.3)	71 (16.7)	29 (12.9)	
Hemo-or pneumothorax requiring drainage					
Yes	144 (17.2)	72 (31.2)	43 (10.1)	29 (12.9)	< 0.001
No	735 (82.7)	159 (68.8)	381 (89.9)	195 (87.1)	
Unknown	5 (0.1)				
Flail chest					
No	22 (2.5)	223 (95.3)	412 (98.1)	211 (98.6)	0.044
Yes	846 (95.7)	11 (4.7)	8 (1.9)	3 (1.4)	
Unknown	16 (1.8)				

### Outcomes

Eighty-eight patients (10.0%) died during hospital admission. Eighty-four patients (9.5%) developed pneumonia. Ninety-eight patients required tracheal intubation and mechanical ventilation (11.1%), of whom 28 (3.2%) of patients were intubated due to delayed respiratory insufficiency. Two hundred thirteen patients (24.1%) were transferred to the ICU straight from the Emergency Department and 43 patients (4.9%) were admitted to the ICU at a later moment from the clinical ward due to delayed respiratory problems. Of all patients transferred to the ICU, only 59 patients (24.8%) had none or minor extra-thoracic injuries. More specifically, of the patients admitted to the ICU, 93 (39.1%) had severe or worse head injuries. In addition, in those requiring intubation and mechanical ventilation, severe or worse head injury was present in 63 cases (64.3%).

Median hospital length of stay was 8 days (P_25_–P_75_ 4–14 days). For those patients admitted to the ICU, median length of stay in ICU was 4 days (P_25_–P_75_ 2–10 days). Five hundred eighteen patients (57.7%) were discharged directly to their prior housing facility after discharge.

### Factors associated with in-hospital death

Patient and injury characteristics associated with in-hospital mortality are listed in Table 3. Mortality was higher in the level-1 trauma center than in level 2 and 3 hospitals. Moreover, increasing age and pre-existing conditions such as cardiac disease and COPD were associated with an increased probability of in-hospital mortality. Injury-specific characteristics such as the number of rib fractures, the need for chest-tube drainage and the severity of thoracic and extra-thoracic injuries are also predicted in hospital death. In patients with four or more rib fractures without or with only minor or moderate extra-thoracic injuries, in-hospital mortality was 7.4%. In comparison, mortality in patients with four or more rib fractures and severe or worse extra-thoracic injuries was 23.3% (*p* = 0.002). In patients with a limited number of rib fractures (< 4) and none or minor extra-thoracic injuries, in-hospital mortality was 3%. In a multivariable analysis, the model with the best fit was obtained after including the following variables in the model: age, presence of COPD GOLD 2 or worse, presence of cardiac disease, presence and severity of head, abdominal, and spinal injury by AIS, number of rib fractures and need for chest tube drainage (Table 4). The explained variance of the model was 21%.

**Table 3:** Univariable analysis of factors associated with in-hospital death and pneumonia

	Number of patients dead (*n* = 88)	Odds ratio (95% CI)	*p* value	Number of patients with pneumonia (*n* = 84)	Odds ratio (95% CI)	*p* value
Age						
65–80	43 (7.4%)			59 (10.4%)		
81–90	35 (14.9%)	1.4 (0.7–2.7)	0.37	20 (8.6%)	1.0 (0.6–1.9)	0.88
≥ 91	10 (14.5%)	2.3 (13 − 4.5)	0.008	5 (7.5%)	0.9 (0.5–1.6)	0.68
Gender						
Male	48 (9.3%)			64 (12.7%)		
Female	40 (10.8)	1.2 (0.8–1.8)	0.46	20 (5.5%)	0.4 (0.2–0.7)	< 0.001
History of cardiac disease						
No	53 (7.5%)			67 (9.6%)	1.2 (0.7–2.1)	0.562
Yes	22 (15.3%)	2.2 (1.3–3.8)	0.003	16 (11.2%)		
Unknown	13			1		
History of COPD GOLD 2 or worse/any grade^a^						
No	69 (8.3%)			61 (8.1%)		
Yes	6 (25%)	3.7 (1.4–9.6)	0.007	22 (23.7%)	3.5 (2.0–6.0)	< 0.001
Unknown	13			1		
Number of rib fractures						
Single	12 (7.1%)			7 (4.3%)		
2–3	19 (5.6%)	0.8 (0.4–1.6)	0.49	27 (8.0%)	1.9 (0.8–4.6)	0.12
≥ 4	31 (13.4%)	2.0 (1.0–4.0)	0.051	33 (14.0%)	3.4 (1.5–7.9)	0.005
Multiple NFS	26 (18.2%)	2.9 (1.4–6.0)	0.004	17 (12.8%)	3.8 (1.5–9.2)	0.004
Need for CTD						
No	56 (7.6%)			57 (7.9%)		
Yes	29 (20.1%)	3.1 (1.9–5.0)	< 0.001	27 (19.0%)	2.5 (1.7–4.5)	< 0.001
Thoracic injuries						
Minor	9 (6.7%)			6 (4.6%)		
Moderate	16 (5.2%)	0.7 (0.3–1.8)	0.77	23 (7.5%)	1.7 (0.7–4.3)	0.26
Severe or worse	63 (14.3%)	2.3 (1.1–4.8)	0.02	55 (12.7%)	3.0 (1.3–7.2)	0.01
Extra-thoracic injuries						
None or minor	25 (5.8%)			31 (7.3%)		
Moderate	16 (7.8%)	1.4 (0.7–2.6)	0.36	11 (5.5%)	0.7 (0.4–1.5)	
Severe or worse	47 (18.8%)	3.7 (2.2–6.3)	< 0.001	42 (17.1%)	2.6 (1.6–4.3)	< 0.001
Head injury						
None or minor	64 (8.3%)			53 (8.0%)		
Moderate	11 (16.9%)	0.4 (0.1–1.6)	0.18	6 (9.7%)	1.2 (0.5-3.0)	0.64
Severe or worse	13 (28.9%)	3.4 (2.1–5.5)	< 0.001	25 (17.5%)	2.4 (1.5–4.1)	0.001
Spinal injury						
None or minor	53 (7.9%)			64 (8.4%)		
Moderate	2 (3.1%)	2.6 (1.0-6.6)	0.04	12 (18.8%)	2.5 (1.3–4.9)	0.008
Severe or worse	33 (22.6%)	6.2 (2.0-19.5)		8 (18.2%)	2.4 (1.1–2.4)	0.32
Abdominal injury						
None or minor	77 (9.1%)			73 (8.8%)		
Moderate	6 (20.7%)	2.6 (1.0-6.6)	0.44	7 (24.1%)	3.3 (1.4-8.0)	0.008
Severe or worse	5 (38.5%)	6.2 (1.9–19.5)	0.002	4 (30.8%)	4.6 (1.4–15.3)	0.013
Level of admitting hospital						
Level 1	51 (21.7%)			38 (16.3%)		
Level 2	25 (5.9%)	0.2 (0.1–0.4)	< 0.001	31 (7.5%)	0.4 (0.3–0.7)	0.001
Level 3	12 (5.4%)	0.2 (0.1–0.4)	< 0.001	15 (6.8%)	0.4 (0.2–0.7)	0.002

*CTD* chest tube drainage, *CI* confidence interval

^a^COPD GOLD 2 or worse for in-hospital death, any grade COPD for pneumonia

**Table 4:** Adjusted odds ratios, 95% confidence intervals and *p* values for the association between patient characteristics, pattern of injury and in-hospital mortality

Independent variable	Odds ratio	95% Confidence interval	*p* value
Age (years)			
65–80	ref		
81–90	1.4	0.6–3.2	0.44
≥ 91	3.4	1.5–7.6	0.003
History of cardiac disease (Yes)	2.6	1.4–4.7	0.003
History of COPD GOLD 2 or more	1.3	1.4–12.7	0.01
Number of rib fractures	2.087	1.249–3.489	0.005
1	Ref		
2–3	0.9	0.4–2.0	0.78
> 3	1.2	0.6–2.9	0.56
Multiple unspecified	2.6	1.1–6.0	0.03
Severity of head injury (AIS)			
None or minor	Ref		
Moderate	0.6	0.12–2.4	0.42
Severe or worse	3.5	1.9–6.4	< 0.001
Severity of abdominal injury (AIS)			
None or minor	ref		
Moderate	2.4	0.8–7.4	0.13
Severe or worse	6.8	1.8–25.4	0.004
Severity of spine injury (AIS)			
None or minor	Ref		
Moderate	1.6	0.7–3.9	0.30
Severe or worse	4.6	1.9–11.2	0.001
Need for chest-tube drainage (yes)	2.0	1.1–3.7	0.03

*AIS* Abbreviated Injury Score, *ISS* Injury Severity Score

### Factors associated with pneumonia

Patient and injury characteristics associated with pneumonia are listed in Table 3. While increasing age did not increase the probability of pneumonia, male patients had a higher risk of pneumonia when compared to female patients. Pre-existing chronic obstructive pulmonary disease was associated with a 23.7% chance of developing pneumonia. Injury-specific characteristics such as the number of rib fractures, need for chest tube drainage, and the severity of intra-thoracic and extra-thoracic injuries were also associated with an increased probability of developing pneumonia. In patients with four or more rib fractures without or with minor or moderate extra-thoracic injuries, 6.9% of patients developed pneumonia. In comparison, of 89 patients with four or more rib fractures and severe or worse extra-thoracic injuries 23 patients (25.8%) developed pneumonia (*p* < 0.001). In patients with none or minor extra-thoracic injuries and 1–3 rib fractures, the rate of pneumonia was 5.6%. In the multivariable analysis, the model with the best fit was obtained after including the following variables in the model: female gender, presence of COPD, number of rib fractures, need for chest tube drainage, and presence and severity of head and abdominal injury by AIS (Table 5). The explained variance of the model was 17%.

**Table 5:** Adjusted odds ratios, 95% confidence intervals and *p* values for the association between patient characteristics, pattern of injury and pneumonia

Independent variable	Odds ratio	95% Confidence interval	*p* value
Gender (male)	2.4	1.4–4.2	0.002
History of COPD (any grade)	3.9	2.2–7.1	< 0.001
Number of rib fractures			
1			
2–3	1.8	0.8–4.4	0.18
> 3	2.6	1.1–6.5	0.03
Multiple unspecified	2.8	1.1–7.4	0.03
Severity of head injury (AIS)	1.356	1.163–1.581	< 0.001
None or minor			
Moderate	1.7	0.7–4.3	0.28
Severe or worse	2.5	1.4–4.3	0.002
Severity of abdominal injury (AIS)			
None or minor			
Moderate	2.2	0.8–6.1	0.14
Severe or worse	7.3	2.0–26.6	0.003
Need for chest tube drainage (Yes)	1.9	1.1–3.4	0.02

*AIS* Abbreviated Injury Score

## Discussion

This multicenter observational study aimed to identify risk factors for pneumonia and death in a large cohort of elderly patients with traumatic rib fractures. In addition, patterns of injury that may influence outcome and further treatment were investigated. Almost half of patients sustained their injuries after a fall from standing height. About a quarter of patients had four or more rib fractures. Moderate or worse extra-thoracic injuries were present in more than half of patients. Increasing age, comorbidity, the number of rib fractures and the severity of extra-thoracic injuries were all associated with an increased risk of pneumonia and in-hospital death, which occurred in about 10% of patients.

Mortality rates up to 22% have been reported in elderly patients with multiple rib fractures [2]. As our data show, mortality rates differ dramatically depending on the type of population that is studied. In the current study, patients admitted to level-3 trauma centers had a mortality rate of 5.4% while patients admitted to level-1 hospitals had a mortality rate of almost 22%. The risk factors for pneumonia and death that were identified in this study have been identified by others as well [4]. Increasing age and the presence of cardiopulmonary comorbidities are well-known risk factor for in-hospital mortality in any trauma patient [5]. A recent study by Harrington et al. including patients from both level-1 centers as well as lower level trauma centers found a mortality rate of 4.9%. Apart from injury severity, age and the presence of congestive heart failure were independently associated with in-hospital mortality [6].

Another frequently reported risk factor for pneumonia and death is the number of rib fractures. In our study—like many others—the presence of four or more rib fractures was independently associated with an increased risk of pneumonia and death. A recent study by Shulzhenko et al. reported a much higher threshold (eight or more fractured ribs) for an elevated risk of death [7]. One of the most likely reasons for this is the increased use of CT scanning in trauma patients, resulting in a higher rate of detected rib fractures when compared to conventional chest radiographs creating a potential detection bias.

When considering these risk factors, the validity of our statistical models should be taken into consideration. The statistical models we created to predict pneumonia and death had a poor predictive value with regard to the explained variance in pneumonia and death (17 and 21%, respectively). This suggests that other important predictors of mortality and pneumonia were not captured in the current study. So-called frailty indices—a composite measure for physical activities, nutritional status, social activities, cognitive performance and overall health status—are validated tools for assessing functional status in the elderly and have been shown to predict outcomes in a variety of surgical diseases, including trauma [8, 9]. A recent study by Joseph et al. showed that frailty was a better predictor of outcomes in the aging trauma patient when compared to age alone. Patients with frailty were more likely to have in-hospital complications and had an increased chance of dying or being discharged to a nursing facility when compared to non-frail patients [10]. While we did not have these data available, further research on geriatric trauma should definitively focus on these frailty indices.

The increasing rate of elderly patients admitted with rib fractures as well as the rate of adverse events in the current population results in a considerable use of resources with regard to ICU and hospital admission. Therefore, tailored treatment aimed at fast recovery and discharge to the previous housing facility with a low probability of adverse events a extremely important.

While oral, intravenous or epidural analgesics combined with physical therapy are currently the gold standard for the treatment of rib fractures, specific analgesic therapies may not always be possible (e.g. epidural analgesics and oral anticoagulants) or may even be harmful (morphine induced delirium) in the elderly population [11, 12]. Encouraged by favorable results with regard to ventilator days and risk of complications after surgical rib fixation in patients with flail chest as shown in the randomized trial by Marasco et al., some have advocated surgical rib fixation in the elderly patient with multiple (non flail chest) rib fractures too [13, 14]. A recent retrospective case control study by Fitzgerald et al. supports this strategy; surgical rib fixation was associated with lower rates of pneumonia, respiratory readmissions in ICU and death [15, 16].

Despite the promising results, the current study shows that not all patients with multiple rib fractures may be candidates for this approach: spinal injuries—which were present in 8% of patients—may preclude proper positioning of these patients for surgical rib fixation. Traumatic brain injury (TBI, which was present in 22% of patients) may require prolonged intubation and mechanical ventilation, and significantly impacts on mortality and may, therefore, decrease the potential benefits of surgical rib fixation with regard to ICU and ventilator times, (ventilator-associated) pneumonia and mortality.

Apart from the risk of selection bias with regard to the number of rib fractures, this study has several other limitations. First, the use of registry data has some important drawbacks, such as the risk of misclassification by AIS, under- or overestimation of the number of rib fractures and the risk of duplicate patients. In addition, apart from data on the use of epidural anesthesia, data on the type and timing of analgesia, and the exact frequency and intensity of physical therapy were not available, which may have a considerable influence on patient outcomes. At last, we did not have data available with regard to some important patient characteristics, such as smoking status, pre-injury cognitive status and housing facility. As stated earlier, these may very well be important determinants of outcome as well.

In conclusion, pneumonia and death occur in about 10% of elderly patients with rib fractures. Comorbidities and extra-thoracic injuries are common and should be considered when choosing between different treatment options in the elderly patient with rib fractures.
